# Trends in Outbreak-Associated Cases of COVID-19 — Wisconsin, March–November 2020

**DOI:** 10.15585/mmwr.mm7004a2

**Published:** 2021-01-29

**Authors:** Ian W. Pray, Anna Kocharian, Jordan Mason, Ryan Westergaard, Jonathan Meiman

**Affiliations:** ^1^Wisconsin Department of Health Services; ^2^CDC COVID-19 Response Team; ^3^Epidemic Intelligence Service, CDC; ^4^School of Medicine and Public Health, University of Wisconsin—Madison.

During September 3–November 16, 2020, daily confirmed cases of coronavirus disease 2019 (COVID-19) reported to the Wisconsin Department of Health Services (WDHS) increased at a rate of 24% per week, from a 7-day average of 674 (August 28–September 3) to 6,426 (November 10–16) (*1*). The growth rate during this interval was the highest to date in Wisconsin and among the highest in the United States during that time (*1*). To characterize potential sources of this increase, the investigation examined reported outbreaks in Wisconsin that occurred during March 4–November 16, 2020, with respect to their setting and number of associated COVID-19 cases.

Outbreaks were defined as the occurrence of two or more confirmed COVID-19 cases* among persons who worked or lived together or among persons who attended the same facility or event, did not share a household, and were identified within 14 days of each other (by symptom onset date or sample collection date). During March 4–November 16, local and tribal health departments in Wisconsin reported suspected COVID-19 outbreaks to WDHS using established reporting criteria^†^; 5,757 reported outbreaks meeting the outbreak definition were included in the analysis. Confirmed cases of COVID-19 that were linked^§^ to these outbreaks were analyzed by symptom onset date (or sample collection date), the reported setting^¶^ of the associated outbreak or outbreaks during three periods: before and during Wisconsin’s Safer At Home order** (March 4–May 12), summer and return-to-school (May 13–September 2), and the exponential growth phase^††^ (September 3–November 16). This activity was reviewed by CDC and was conducted in a manner consistent with applicable federal law and CDC policy.^§§^

A total of 57,991 confirmed cases of COVID-19 were linked to 5,757 outbreaks during March 4–November 16, accounting for 18.3% of 316,758 confirmed cases in Wisconsin during this period (Table). Overall, outbreaks at long-term care facilities (26.8%), correctional facilities (14.9%), and colleges or universities (15.0%) accounted for the largest numbers of outbreak-associated cases in Wisconsin. Before and during Wisconsin’s Safer At Home order, 4,552 outbreak-associated cases were linked to 507 reported outbreaks. Outbreaks at manufacturing or food processing facilities (2,146 cases; 47.1%) and long-term care facilities (1,324 cases; 29.1%) accounted for the majority of outbreak-associated cases during this period (Figure). During May 13–September 2, a total of 13,506 cases were linked to 2,444 outbreaks. Long-term care facilities (2,850 cases; 21.1%) and manufacturing or food processing facilities (2,672 cases; 19.8%) continued to account for the largest number of outbreak-associated cases during this period. However, a variety of other settings including restaurants and bars (1,633 cases; 12.1%) and other workplaces (1,320 cases; 9.8%) accounted for an increasing proportion of outbreak-associated cases until mid-August, when a sharp increase in college- and university-associated outbreaks was observed (1,739 cases; 12.9%). Beginning on September 3, COVID-19 cases in Wisconsin increased exponentially overall and within outbreak settings. During this phase of increasing community transmission, 39,933 cases were associated with 3,861 reported outbreaks, which accounted for 16.7% of 239,629 confirmed cases in Wisconsin. Among outbreak-associated cases, 11,386 (28.5%) were associated with long-term care facilities, 7,397 (18.5%) with correctional facilities, 7,178 (18.0%) with colleges or universities, and 5,703 (14.3%) with schools or child care facilities. During this period of exponential growth, the number of cases associated with long-term care and correctional facilities increased by an average of 24% and 23% per week, respectively.

**TABLE:** Laboratory-confirmed COVID-19 cases associated with outbreaks by settings, and period of the COVID-19 response — Wisconsin, March–November 2020

Outbreak setting	No. (%)			
	Mar 4–May 12	May 13–Sep 2	Sep 3–Nov 16	Total
Long-term care facility	1,324 (29.1)	2,850 (21.1)	11,386 (28.5)	**15,529 (26.8)**
College or university	36 (0.8)	1,739 (12.9)	7,178 (18.0)	**8,689 (15.0)**
Correctional facility	307 (6.7)	964 (7.1)	7,397 (18.5)	**8,661 (14.9)**
K–12 school or child care facility	10 (0.2)	461 (3.4)	5,704 (14.3)	**6,145 (10.6)**
Food production or manufacturing facility*	2,146 (47.1)	2,672 (19.8)	3,631 (9.1)	**8,436 (14.5)**
Restaurant or bar	82 (1.8)	1,633 (12.1)	917 (2.3)	**2,628 (4.5)**
Retail or public establishment	45 (1.0)	814 (6.0)	1,053 (2.6)	**1,902 (3.3)**
Event or gathering	39 (0.9)	761 (5.6)	1,113 (2.8)	**1,885 (3.3)**
Health care facility	115 (2.5)	444 (3.3)	1,214 (3.0)	**1,768 (3.0)**
Other group housing facility	249 (5.5)	352 (2.6)	781 (2.0)	**1,375 (2.4)**
Other workplaces^†^	292 (6.4)	1,320 (9.8)	1,985 (5.0)	**3,585 (6.2)**
Other settings	48 (1.1)	794 (5.9)	1,424 (3.6)	**2,222 (3.8)**
**Total^§^**	**4,552**	**13,506**	**39,933**	**57,991**

**Abbreviations:** COVID-19 = coronavirus disease 2019; K–12 = kindergarten through grade 12.

* Includes food production and processing, meat processing, manufacturing facilities, and distribution or warehouse facilities.

^†^ Includes agriculture, farming, forestry, construction, contracting, office or other indoor workplace, public safety, transportation, and utilities.

^§^ Some cases were associated with multiple outbreak settings because multiple epidemiologic linkages were identified during the outbreak investigation; thus, the sum of all categories exceeds the total number of cases listed for each period.

**FIGURE:**
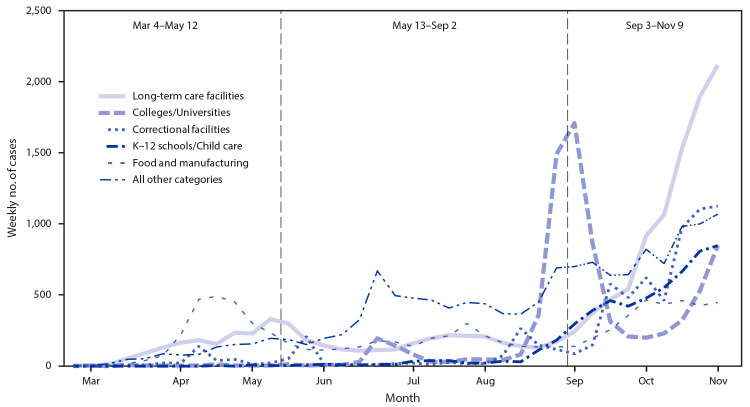
Trends* in the number of laboratory-confirmed COVID-19 cases associated with outbreaks, by setting^†^ and period of the COVID-19 response — Wisconsin, March–November 2020 **Abbreviations:** COVID-19 = coronavirus disease 2019; K–12 = kindergarten through grade 12. * Data from November 10–16, 2020 are not displayed in the figure, but are represented in the counts that appear in text and footnotes. ^†^ All other categories includes restaurant or bar (4.2%), retail or other public establishment (3.1%), event or gathering (3.0%), health care facility (2.8%), other group housing (2.2%), other workplaces (5.7%), and other settings (3.5%).

## Discussion

The majority of outbreak-associated COVID-19 cases in Wisconsin occurred in long-term care facilities, correctional facilities, and colleges and universities; however, various settings were affected by COVID-19 outbreaks over the course of March–November 2020. During Wisconsin’s Safer At Home order, outbreaks were concentrated in manufacturing and food processing facilities, which continued to operate as essential businesses under the statewide order. This aligned with national data showing a high incidence of COVID-19 outbreaks at meat processing facilities across the United States during this time, including among beef and pork processing facilities in Wisconsin (*2*). During early summer (June–July), outbreaks continued to occur in long-term care facilities and manufacturing and food processing facilities; restaurants and bars, other workplaces, events, and other public establishments were increasingly reported as outbreak settings, which might have corresponded to fewer restrictions on social gatherings and decreased risk perception among some groups during this period (*3*).

In late August, a rapid increase in cases associated with outbreaks at colleges and universities in Wisconsin occurred, correlating with return to campus for many of these institutions. This pattern was consistent with national trends for COVID-19 among young adults aged 18–22 years (*4*) and corresponded with outbreaks observed at colleges and universities in other states during this time (*5*). In Wisconsin, the college and university surge occurred at the beginning of a period of increasing community transmission, which was characterized by exponential growth in COVID-19 incidence across the state and a surge of outbreaks in high-risk congregate settings such as long-term care facilities and correctional facilities. The extent to which COVID-19 outbreaks on college and university campuses led to increased community transmission and subsequent outbreaks in other high-risk congregate settings could not be directly assessed in this investigation. Nonetheless, the temporal correlation observed builds on prior evidence of increased incidence of COVID-19 among U.S. counties where in-person university instruction occurred in August 2020 (*6*), suggesting that outbreaks on college and university campuses could represent early indicators of community transmission and should be prioritized for surveillance and mitigation planning.

The findings in this report are subject to at least three limitations. First, an absence of reported outbreaks in some settings should not be interpreted as an absence of COVID-19 cases in these settings, because local and tribal health departments in Wisconsin directed limited resources to investigate outbreaks in high-risk congregate settings. Therefore, lower-risk settings might be underrepresented. Second, local and tribal health departments could not verify epidemiologic linkages for all cases in all outbreaks, and some outbreak-associated cases could have occurred in other settings not represented in this analysis. Finally, use of these surveillance data alone cannot determine whether outbreaks in one setting are directly responsible for increases in community transmission or outbreaks in other settings; more detailed epidemiologic or genomic data are needed to explore whether such temporal correlations are causally related.

Examining trends in COVID-19 outbreaks over time provides an important indicator of COVID-19 incidence across sectors in response to changing behaviors and policies. State, local, and tribal health departments should continue to collect and report such information, particularly among highly affected sectors such as long-term care facilities and correctional facilities. Further, given the importance of college and university outbreaks as potential early indicators of outbreaks in other settings, colleges and universities should work with public health officials to strengthen surveillance and mitigation strategies to prevent COVID-19 transmission.

**Summary**

**What is already known about this topic?**

COVID-19 incidence grew sharply in Wisconsin during September–November 2020; however, the underlying cause of this rapid growth is unknown.

**What is added by this report?**

An examination of COVID-19 outbreaks in Wisconsin showed that cases linked to outbreaks on college and university campuses increased sharply in August 2020 and were followed by outbreaks in other high-risk congregate settings. Overall, outbreaks at long-term care facilities (26.8%), correctional facilities (14.9%), and colleges or universities (15.0%) accounted for the largest numbers of outbreak-associated cases in Wisconsin.

**What are the implications for public health practice?**

COVID-19 surveillance and mitigation planning should be prioritized for highly affected settings such as long-term care facilities, correctional facilities, and colleges and universities, which could represent early indicators of broader community transmission.

## Footnotes

* Confirmed cases of COVID-19 were defined according to Council of State and Territorial Epidemiologists 2020 interim case definition requiring detection of SARS-CoV-2 RNA using a molecular amplification test (https://wwwn.cdc.gov/nndss/conditions/coronavirus-disease-2019-covid-19/case-definition/2020/08/05/).

† Suspected outbreaks were reportable to the Wisconsin Department of Health Services if two or more patients with confirmed COVID-19 (one or more patients for long-term care facilities) had symptom onset dates (or sample collection dates) within 28 days, worked or lived together, or attended the same facility or event, and did not share a household. For colleges and universities, some local and tribal health departments reported outbreaks for cases among students and faculty who attended the same institution. Only reported outbreaks that met additional inclusion criteria (two or more confirmed cases with symptom onset or sample collection within 14 days) were included in the analysis.

§ Cases were linked to multiple outbreaks if multiple associations were identified and determined to be epidemiologically linked to multiple settings during the case investigation interview.

¶ Outbreak setting categories included long-term care facilities, correctional or detention facilities, kindergarten through grade 12 schools or child care facilities, colleges or universities, manufacturing or food processing facilities, restaurants or bars, retail or other public establishments, events or gatherings, health care facilities, other group housing, other workplaces, and other settings.

**
https://evers.wi.gov/Documents/COVID19/EMO28-SaferAtHome.pdf

†† The beginning of exponential growth phase (September 3) marked the date on which the weekly average number of new confirmed cases began to increase exponentially after declining for 5 consecutive weeks (July 26–September 2). Daily and weekly confirmed cases in Wisconsin are available (https://www.dhs.wisconsin.gov/covid-19/cases.htm).

§§ Activity was determined to meet the requirements of public health surveillance as defined in 45 CFR 46.102(l)(2).
