# *Erratum:* Vol. 70, No. 4

**DOI:** 10.15585/mmwr.mm7005a5

**Published:** 2021-02-05

In the report “Trends in Outbreak-Associated Cases of COVID-19 — Wisconsin, March–November 2020,” on page 114, the third sentence of the second paragraph should have read “Confirmed cases of COVID-19 that were linked^§^ to these outbreaks were analyzed by symptom onset date (or sample collection date) **and** the reported setting^¶^ of the associated outbreaks during three periods: before and during Wisconsin’s Safer At Home order** (March 4–May 12), summer and return-to-school (May 13–September 2), and the exponential growth phase^††^ (September 3–November 16).”

